# Spectral properties of an impulsive Sturm–Liouville operator

**DOI:** 10.1186/s13660-018-1781-0

**Published:** 2018-07-27

**Authors:** Elgiz Bairamov, Ibrahim Erdal, Seyhmus Yardimci

**Affiliations:** Department of Mathematics, Faculty of Science, Ankara University, Ankara, Turkey

**Keywords:** 34B37, 34L05, 34L25, 34L40, 34B09, Impulsive operators, Sturm–Liouville equations, Eigenvalues, Spectral singularities, Scattering function

## Abstract

This work is devoted to discuss some spectral properties and the scattering function of the impulsive operator generated by the Sturm–Liouville equation. We present a different method to investigate the spectral singularities and eigenvalues of the mentioned operator. We also obtain the finiteness of eigenvalues and spectral singularities with finite multiplicities under some certain conditions. Finally, we illustrate our results by a detailed example.

## Introduction

In this paper, we consider the Sturm–Liouville equation on the semi axis

1.1 $$ -y''+q ( x ) y=\lambda^{2}\rho ( x ) y,\quad x\in{[0,\infty)}, $$

with boundary condition

1.2 $$ y(0)=0, $$

where *λ* is a spectral parameter, and *ρ* is the density function. There is a comprehensive literature on the spectral theory of boundary value problem (1.1)–(1.2) for $\rho=1$. In particular, the spectral analysis of the problem having discrete and continuous spectrum was begun by Naimark [1] for $\rho=1$. He proved that some poles of the resolvent kernel are not the eigenvalues of the operator. He also showed that those poles, which are called spectral singularities by Schwartz [2], are a mathematical obstruction for the completeness of the eigenvectors and are embedded in the continuous spectrum. Pavlov [3] established the dependence of the structure of the spectral singularities of the differential operator on the behavior of the potential function at infinity. So far, a large number of problems related to the spectral analysis of differential and some other types of operators with spectral singularities have been investigated [4–10]. As is well known, the Sturm–Liouville equation (1.1) has a bounded solution satisfying the condition

$$\lim_{x \rightarrow\infty} e(x,\lambda)e^{-i\lambda x}=1, $$

where

$$\lambda\in\mathbb{\overline{C}}_{+}:= \lbrace \lambda\in\mathbb {C}: \operatorname{Im} \lambda\geq0 \rbrace, $$

and $e ( x,\lambda ) $ is the Jost solution of (1.1) and has the integral representation

1.3 $$ e(x,\lambda)=e^{i\lambda x}+ \int_{x}^{\infty}K(x,t)e^{i\lambda t}\,dt,\quad\lambda\in \mathbb{\overline{C}}_{+}, $$

under the condition

1.4 $$ \int_{0}^{\infty}x \bigl\lvert q(x) \bigr\rvert \,dx< \infty, $$

where $K(x,t) $ is defined by the potential function *q* [11, 12].

Furthermore, boundary value problems with discontinuities inside an interval have great interest in mathematical physics and quantum mechanics. To solve interior discontinuities, some extra conditions are imposed on the discontinuous point, which are often called interface conditions, point interactions, transmission conditions, and impulsive conditions. The theory of impulsive differential equations were studied in applied mathematics in detail [13, 14]. A great number of authors studied the spectral theory of impulsive differential equations [15–18]. Moreover, the physical meaning and potential applications of spectral singularities of impulsive differential equations have been understood quite recently [19, 20]. Especially in [21], the author provided the physical meanings of eigenvalues and spectral singularities of the Schrödinger equation at a single point. Such problems have been widely studied for impulsive differential operators on the whole axis.

In this work, we are concerned with the impulsive Sturm–Liouville operator on the semi axis. The density function *ρ* and impulsive condition make the spectral analysis of operator quite difficult, but by determining a transfer matrix we can obtain some spectral properties.

## Statement of the problem

Let us introduce the Sturm–Liouville operator *L* in $L^{2}[0,\infty)$ generated by the equation

2.1 $$ -y''+q ( x ) y=\lambda^{2}\rho ( x ) y,\quad x\in{[0,x_{0})}\cup ( x_{0},\infty ), $$

with the boundary condition

2.2 $$ y(0)=0 $$

and the impulsive condition

2.3 $$ \begin{bmatrix} y ( x_{0}^{+} ) \\ y' ( x_{0}^{+} ) \end{bmatrix} =B \begin{bmatrix} y ( x_{0}^{-} ) \\ y' ( x_{0}^{-} ) \end{bmatrix} ,\qquad B= \begin{bmatrix} \alpha_{1} & \alpha_{2}\\ \alpha_{3} & \alpha_{4} \end{bmatrix} , $$

where $\alpha_{1}$, $\alpha_{2}$, $\alpha_{3}$, $\alpha_{4}$ are complex numbers such that $\det B\neq0 $, *λ* is a spectral parameter, $x_{0}$ is a positive real constant, and the real-valued potential function *q* satisfies the condition

2.4 $$ \int_{0}^{\infty}x \bigl\lvert q(x) \bigr\rvert \,dx< \infty. $$

The density function *ρ* has the form

2.5 $$ \rho ( x ) = \textstyle\begin{cases} \beta^{2},& 0\leq x< x_{0},\\ 1,& x>x_{0}, \end{cases} $$

where $\beta\in\mathbb{R}\backslash(-1,1) $.

Note that $x=x_{0}$ is the impulsive point of problem (2.1)–(2.3), and the matrix *B* is used to continue the solution of (2.1) from $[ 0,x_{0} ) $ to $(x_{0},\infty ) $.

Furthermore, we denote the solutions of equation (2.1) by $y_{-}$ and $y_{+}$, respectively:

$$\textstyle\begin{cases} y_{-} ( x ) :=y ( x ) ,& 0\leq x< x_{0},\\ y_{+} ( x ) :=y ( x ) ,& x>x_{0}. \end{cases} $$

It is known that $S ( x,\lambda^{2} ) $ and $C ( x,\lambda ^{2} ) $ are the fundamental solutions of (2.1) in the interval $[ 0,x_{0} ) $ fulfilling the conditions

$$S \bigl( 0,\lambda^{2} \bigr) =0,\qquad S' \bigl( 0, \lambda^{2} \bigr) =1 $$

and

$$C \bigl( 0,\lambda^{2} \bigr) =1,\qquad C' \bigl( 0, \lambda^{2} \bigr) =0, $$

respectively. The solutions $S ( x,\lambda^{2} ) $ and $C ( x,\lambda^{2} ) $ can be expressed in the form

2.6 $$ S \bigl( x,\lambda^{2} \bigr) =\frac{\sin\lambda\beta x}{\lambda\beta}+ { \int_{0}^{x}} P ( x,t ) \frac{\sin\lambda\beta t}{\lambda\beta}\,dt $$

and

2.7 $$ C \bigl( x,\lambda^{2} \bigr) =\cos\lambda\beta x+ { \int_{0}^{x}} Q ( x,t ) \cos\lambda\beta t\,dt , $$

where the kernel functions $P ( x,t ) $ and $Q ( x,t ) $ can be given in terms of the potential function *q* [11]. Besides, $S ( x,\lambda^{2} ) $ and $C ( x,\lambda^{2} ) $ are entire functions of *λ*, and

$$W \bigl[ S \bigl( x,\lambda^{2} \bigr) ,C \bigl( x, \lambda^{2} \bigr) \bigr] =-1,\quad\lambda\in\mathbb{C}, $$

where $W [ y_{1},y_{2} ] $ denotes the Wronskian of the solutions $y_{1}$ and $y_{2}$ of equation (2.1).

On the other hand, (2.1) admits another solution

2.8 $$ e ( x,-\lambda ) =e^{-i\lambda x}+ { \int_{x}^{\infty}} K ( x,t ) e^{-i\lambda t}\,dt,\quad \lambda\in\overline{C}_{-}, $$

in $(x_{0},\infty ) $, fulfilling the asymptotic condition

$$\lim_{x \rightarrow\infty} e(x,-\lambda)e^{i\lambda x}=1, $$

where $\lambda\in\mathbb{\overline{C}}_{-}:= \lbrace \lambda\in \mathbb{C}:\operatorname{Im} \lambda\leq0 \rbrace $. Obviously,

$$W \bigl[ e ( x,\lambda ),e ( x,-\lambda ) \bigr] =-2i\lambda,\quad\lambda\in \mathbb{R}\backslash \{ 0 \} . $$

Also, $K(x,t) $ satisfies

2.9 $$\begin{aligned} &\bigl\vert K(x,t) \bigr\vert \leq c\sigma \biggl( \frac{x+t}{2} \biggr), \end{aligned}$$

2.10 $$\begin{aligned} &\bigl\vert K_{x}(x,t) \bigr\vert \leq\frac{1}{4} \biggl\vert q \biggl( \frac{x+t}{2} \biggr) \biggr\vert +c\sigma \biggl( \frac{x+t}{2} \biggr), \end{aligned}$$

where $c>0 $ is a constant, and

$$\sigma(x)= \int_{0}^{\infty} \bigl\vert q(t) \bigr\vert \,dt. $$

Now, let $\lambda\in\mathbb{R} \backslash \{ 0 \} $. Using linearly independent solutions of (2.1) in the intervals $[ 0,x_{0} ) $ and $( x_{0},\infty ) $, we can express the general solution of (2.1) by

$$\textstyle\begin{cases} y_{-} ( x,\lambda ) =A_{-}C ( x,\lambda^{2} ) +B_{-}S ( x,\lambda^{2} ) ,&0\leq x< x_{0},\\ y_{+} ( x,\lambda ) =A_{+}e ( x,\lambda ) +B_{+}e (x,-\lambda ) ,& x>x_{0}, \end{cases} $$

where $A_{\pm}$ and $B_{\pm}$ are constant coefficients depending on *λ*. By (1.3), (2.6), (2.7), and (2.8) we get $y_{-} ( x_{0}^{-},\lambda ) $, $y_{+} ( x_{0}^{+},\lambda ) $, $y_{-}' ( x_{0}^{-},\lambda ) $, and $y_{+}' ( x_{0}^{+},\lambda ) $. Then, from the impulsive condition (2.3) we have transfer matrix *M* satisfying

2.11 $$ \begin{bmatrix} A_{+}\\ B_{+} \end{bmatrix} =M \begin{bmatrix} A_{-}\\ B_{-} \end{bmatrix} , $$

where

2.12 $$ M:= \begin{bmatrix} M_{11} & M_{12}\\ M_{21} & M_{22} \end{bmatrix} =N^{-1}BD $$

with

2.13 $$ D:= \begin{bmatrix} C ( x_{0},\lambda^{2} ) & S ( x_{0},\lambda^{2} ) \\ C' ( x_{0},\lambda^{2} ) & S' ( x_{0} ,\lambda^{2} ) \end{bmatrix} $$

and

2.14 $$ N:= \begin{bmatrix} N_{11} & N_{12}\\ N_{21} & N_{22} \end{bmatrix} , $$

where

$$\begin{gathered} N_{11}=e^{i\lambda x_{0}}+ { \int_{x_{0}}^{\infty}} K ( x_{0},t ) e^{i\lambda t}\,dt, \\ N_{12}=e^{-i\lambda x_{0}}+ { \int_{x_{0}}^{\infty}} K ( x_{0},t ) e^{-i\lambda t}\,dt, \\ N_{21}=i\lambda e^{i\lambda x_{0}}-K ( x_{0},x_{0} ) e^{i\lambda x_{0}}+ { \int_{x_{0}}^{\infty}} K_{x} ( x_{0},t ) e^{i\lambda t}\,dt, \\ N_{22}=-i\lambda e^{-i\lambda x_{0}}-K ( x_{0},x_{0} ) e^{-i\lambda x_{0}}+ { \int_{x_{0}}^{\infty}} K_{x} ( x_{0},t ) e^{-i\lambda t}\,dt, \end{gathered} $$

and $K_{x}(x,t):=\frac{\partial}{\partial x}K(x,t) $. Since $\det N=-2i\lambda$, we easily obtain

2.15 $$\begin{aligned}& \begin{aligned}[b] M_{22} ( \lambda ) &=\frac{i}{2\lambda} \bigl\{ -e' ( x_{0},\lambda ) \bigl[ \alpha_{1} S \bigl( x_{0},\lambda^{2} \bigr) +\alpha_{2} S' \bigl( x_{0},\lambda^{2} \bigr) \bigr]\\ &\quad {} +e ( x_{0},\lambda ) \bigl[ \alpha_{3} S \bigl( x_{0},\lambda^{2} \bigr) +\alpha_{4} S' \bigl( x_{0},\lambda^{2} \bigr) \bigr] \bigr\} ,\end{aligned} \end{aligned}$$

2.16 $$\begin{aligned}& \begin{aligned}[b] M_{12} ( \lambda )&=\frac{i}{2\lambda} \bigl\{ e' ( x_{0},-\lambda ) \bigl[ \alpha_{1} S \bigl( x_{0},\lambda^{2} \bigr) +\alpha_{2} S' \bigl( x_{0},\lambda^{2} \bigr) \bigr]\\ &\quad {} -e ( x_{0},-\lambda ) \bigl[ \alpha_{3} S \bigl( x_{0},\lambda ^{2} \bigr) +\alpha_{4} S' \bigl( x_{0},\lambda^{2} \bigr) \bigr] \bigr\} . \end{aligned} \end{aligned}$$

Let us consider any two solutions of (2.1), denoting the coefficients $A_{\pm}$ and $B_{\pm}$ by $A_{\pm}^{\pm}$ and $B_{\pm }^{\pm}$, which are expressed as

2.17 $$ F ( x,\lambda ) = \textstyle\begin{cases} A_{-}^{+}C ( x,\lambda^{2} ) +B_{-}^{+}S ( x,\lambda ^{2} ) ,& 0\leq x< x_{0},\\ A_{+}^{+}e ( x,\lambda ) +B_{+}^{+}e ( x,-\lambda ) ,& x_{0}< x< \infty, \end{cases} $$

and

2.18 $$ G ( x,\lambda ) = \textstyle\begin{cases} A_{-}^{-}C ( x,\lambda^{2} ) +B_{-}^{-}S ( x,\lambda ^{2} ) ,& 0\leq x< x_{0},\\ A_{+}^{-}e ( x,\lambda ) +B_{+}^{-}e ( x,-\lambda ) ,& x_{0}< x< \infty, \end{cases} $$

where $A_{\pm}^{\pm}$ and $B_{\pm}^{\pm}$ are complex coefficients. Let *F* and *G* be associated with the Jost solution of the boundary value problem (2.1)–(2.3) and the boundary condition (2.2), respectively. Then we obtain

2.19 $$ B_{+}^{+}=0,\qquad A_{+}^{+}=1, \qquad A_{-}^{-}=0,\qquad B_{-}^{-}=1. $$

Furthermore, using the impulsive condition (2.3) and (2.11), we get

2.20 $$ A_{-}^{+}=\frac{M_{22}(\lambda)}{\det M},\qquad B_{-}^{+}=-\frac {M_{21}(\lambda)}{\det M}, \qquad A_{+}^{-}=M_{12}( \lambda), \qquad B_{+}^{-}=M_{22}(\lambda) $$

uniquely for the solution *F* and *G*. Clearly, inserting these coefficients into (2.17) and (2.18), we obtain the solutions *F* and *G* satisfying the following asymptotic equations, respectively,

2.21 $$ F ( x,\lambda ) = \textstyle\begin{cases} \frac{M_{22} ( \lambda )}{\det M}C ( x,\lambda^{2} ) -\frac{M_{21} ( \lambda )}{\det M}S ( x,\lambda^{2} ), & x\rightarrow0^{+},\\ e ( x,\lambda ) ,& x\rightarrow\infty, \end{cases} $$

and

2.22 $$ G ( x,\lambda ) = \textstyle\begin{cases} S ( x,\lambda^{2} ), & x\rightarrow0^{+},\\ M_{12} ( \lambda )e ( x,\lambda ) +M_{22} ( \lambda )e ( x,-\lambda ),& x\rightarrow\infty. \end{cases} $$

Now by (2.21) and (2.22) we can give the following lemma.

### Lemma 2.1

*The following equations hold for all* $\lambda\in\mathbb{R} \backslash \{ 0 \} $:

$$\begin{aligned} & W[F,G] ( x,\lambda ) =-2i\lambda M_{22}(\lambda),\quad x \rightarrow \infty, \\ & W[F,G] ( x,\lambda ) =\frac{M_{22}(\lambda)}{\det M},\quad x\rightarrow0^{+}. \end{aligned}$$

Note that, the Wronskian of the solutions of (2.1) in the intervals $[ 0,x_{0} ) $ and $( x_{0},\infty ) $ are independent of *x*, but they are not equal because of the characteristic feature of impulsive differential equations.

Moreover, from (2.15) we understand that $M_{22}$ has an analytic continuation from the real axis to the set $\mathbb{C} _{+}:= \{ \lambda:\lambda\in\mathbb{C}, \;\operatorname{Im} \lambda>0 \} $ and continuous up to the real axis because of analytic properties of solutions $e ( x,\lambda ) $, $e' ( x,\lambda ) $, $S ( x,\lambda^{2} ) $, $S' ( x,\lambda^{2} ) $. By Lemma 2.1 and [22], we have the following.

### Corollary 2.2

*A necessary and sufficient condition to investigate the eigenvalues and spectral singularities of the impulsive Sturm–Liouville operator* *L* *is to investigate the zeros of the function* $M_{22} $.

By (2.15) we have the following representation for $M_{22} $:

2.23 $$\begin{aligned} M_{22} ( \lambda ) =&\frac{i}{2\lambda} \biggl\{ e^{i\lambda x_{0}} \biggl[ -\frac {i\alpha_{1}}{\beta}\sin\lambda\beta x_{0}-i \alpha_{2}\lambda\cos\lambda \beta x_{0}+ \alpha_{2}K ( x_{0},x_{0} ) \cos\lambda\beta x_{0} \\ & {} +\frac{\sin\lambda\beta x_{0}}{\lambda\beta} \bigl( \alpha _{1}K ( x_{0},x_{0} ) -i\alpha_{2}\lambda P ( x_{0},x_{0} ) + \alpha_{2}K ( x_{0},x_{0} ) P ( x_{0},x_{0} ) \bigr) \\ &{} +\alpha_{3}\frac{\sin\lambda\beta x_{0}}{\lambda\beta }+\alpha_{4}\cos \lambda\beta x_{0}+\alpha_{4}P ( x_{0},x_{0} ) \frac{\sin\lambda\beta x_{0}}{ \lambda\beta} \biggr] \\ &{}+ \bigl( \alpha_{1}K ( x_{0},x_{0} ) e^{i\lambda x_{0}}-i\alpha_{1}\lambda e^{i\lambda x_{0}}+ \alpha_{3}e^{i\lambda x_{0}} \bigr) { \int_{0}^{x_{0}}} P ( x_{0},t ) \frac{\sin\lambda\beta t}{\lambda\beta}\,dt \\ &{}+ \bigl( \alpha_{2}K ( x_{0},x_{0} ) e^{i\lambda x_{0}}-i\alpha_{2}\lambda e^{i\lambda x_{0}}+ \alpha_{4}e^{i\lambda x_{0}} \bigr) { \int_{0}^{x_{0}}} P_{x} ( x_{0},t ) \frac{\sin\lambda\beta t}{\lambda\beta}\,dt \\ &{}+ \biggl( \alpha_{3}\frac{\sin\lambda\beta x_{0}}{\lambda\beta}+\alpha _{4}\cos \lambda\beta x_{0}+\alpha_{4}P ( x_{0},x_{0} ) \frac{\sin\lambda\beta x_{0}}{ \lambda\beta} \biggr) { \int_{x_{0}}^{\infty}} K ( x_{0},t ) e^{i\lambda t}\,dt \\ &{} - \biggl( \alpha_{1}\frac{\sin\lambda\beta x_{0}}{\lambda\beta }+\alpha_{2}\cos \lambda\beta x_{0}+\alpha_{2}P ( x_{0},x_{0} ) \frac{\sin\lambda\beta x_{0}}{ \lambda\beta} \biggr) { \int_{x_{0}}^{\infty}} K_{x} ( x_{0},t ) e^{i\lambda t}\,dt \\ &{} +{ \int_{x_{0}}^{\infty}} K_{x} ( x_{0},t ) e^{i\lambda t}\,dt \biggl[ -\alpha_{1} { \int_{0}^{x_{0}}} P ( x_{0},t ) \frac{\sin\lambda\beta t}{\lambda\beta}\,dt -\alpha_{2} { \int_{0}^{x_{0}}} P_{x} ( x_{0},t ) \frac{\sin\lambda\beta t}{\lambda\beta }\,dt \biggr] \\ &{} +{ \int_{x_{0}}^{\infty}} K ( x_{0},t ) e^{i\lambda t}\,dt \biggl[ +\alpha_{3} { \int_{0}^{x_{0}}} P ( x_{0},t ) \frac{\sin\lambda\beta t}{\lambda\beta}\,dt \\ &{} +\alpha_{4} { \int_{0}^{x_{0}}} P_{x} ( x_{0},t ) \frac{\sin\lambda\beta t}{\lambda\beta}\,dt \biggr] \biggr\rbrace . \end{aligned}$$

## Main results

We introduce the sets of spectral singularities and eigenvalues of impulsive operator *L* as

$$\sigma_{ss} ( L ) = \bigl\{ \mu=\lambda^{2}: \operatorname{Im} \lambda=0, \lambda\neq0\text{ and }M_{22} ( \lambda ) =0 \bigr\} $$

and

$$\sigma_{d} ( L ) = \bigl\{ \mu=\lambda^{2}: \operatorname{Im} \lambda>0\text{ and }M_{22} ( \lambda ) =0 \bigr\} , $$

respectively. To study numerical properties of the sets $\sigma _{ss} ( L ) $ and $\sigma_{d} ( L ) $, we investigate the numerical properties of the zeros of $M_{22} $ in $\lambda\in\overline{C}_{+} $.

Now, we define the sets

$$\begin{gathered} S_{1}= \bigl\lbrace \lambda:\lambda\in{C}_{+}, \;M_{22}(\lambda)=0 \bigr\rbrace , \\ S_{2}= \bigl\lbrace \lambda:\lambda\in\mathbb{R}\setminus{ \lbrace 0 \rbrace}, \;M_{22}(\lambda)=0 \bigr\rbrace . \end{gathered} $$

Thus we can rewrite the sets

$$\sigma_{d} ( L ) = \bigl\{ \mu: \mu=\lambda^{2}, \;\lambda \in S_{1} \bigr\} ,\qquad\sigma_{ss} ( L ) = \bigl\{ \mu: \mu= \lambda ^{2},\; \lambda\in S_{2} \bigr\} . $$

### Theorem 3.1

*Under condition* (2.4), *the function* $M_{22}$ *satisfies the following asymptotic equations*:

3.1 $$\begin{aligned}& M_{22}=e^{i\lambda x_{0} ( 1-\beta ) }\frac{\alpha_{2}}{4} \biggl[ 1+O \biggl( \frac{1}{\lambda} \biggr) \biggr],\quad\beta\geq1, \end{aligned}$$

3.2 $$\begin{aligned}& M_{22}=e^{i\lambda x_{0} ( 1+\beta ) }\frac{\alpha_{2}}{4} \biggl[ 1+O \biggl( \frac{1}{\lambda} \biggr) \biggr],\quad\beta\leq-1, \end{aligned}$$

*where* $\alpha_{2}\neq0 $, $\lambda\in\overline {C}_{+}$, *and* $\vert \lambda \vert \rightarrow\infty $.

### Proof

The derivative of the Jost solution $e ( x,\lambda ) $ satisfies the following asymptotic:

3.3 $$ e'(x,\lambda)=e^{i\lambda x}\bigl[i\lambda+O(1) \bigr],\quad x \in[0,\infty),\text{ }\lambda\in\overline {C}_{+},\text{ } \vert \lambda \vert \rightarrow\infty. $$

From (2.15) and (3.3) we can express the function $M_{22} $ for $\lambda\in\overline{C}_{+} $ when $\beta\geq1 $, $\alpha_{2}\neq 0 $, and $\vert \lambda \vert \rightarrow\infty $:

$$\begin{aligned} M_{22}(\lambda) =&e^{i\lambda x_{0} ( 1-\beta ) } \biggl\{ -\frac{ i\alpha_{1}}{2} \biggl[ \frac{e' ( x_{0},\lambda ) e^{-i\lambda x_{0}} }{\lambda} \biggr] \bigl[ S \bigl( x_{0}, \lambda^{2} \bigr) e^{i\lambda\beta x_{0}} \bigr] \\ &{}-\frac{i\alpha_{2}}{2} \biggl[ \frac{e' ( x_{0},\lambda )e^{-i\lambda x_{0}} }{ \lambda} \biggr] \bigl[ S' \bigl( x_{0},\lambda ^{2} \bigr) e^{i\lambda\beta x_{0}} \bigr] +\frac{i\alpha_{3}}{2\lambda } \bigl[ e ( x_{0},\lambda ) e^{-i\lambda x_{0}} \bigr] \bigl[ S \bigl( x_{0},\lambda^{2} \bigr) e^{i\lambda\beta x_{0}} \bigr] \\ &{} +\frac{i\alpha_{4}}{2\lambda} \bigl[ e ( x_{0},\lambda ) e^{-i\lambda x_{0}} \bigr] \bigl[ S' \bigl( x_{0},\lambda ^{2} \bigr) e^{i\lambda\beta x_{0}} \bigr] \biggr\} \\ =&e^{i\lambda x_{o} ( 1-\beta ) } \biggl\{ \frac{\alpha _{1}}{4i\beta} \biggl[ \frac{e^{2i\lambda\beta x_{0}}}{\lambda}- \frac{1}{\lambda }+\frac{1 }{\lambda} \int_{0}^{x_{0}}P ( x_{0},t ) \bigl[ e^{i\lambda \beta ( x_{0}+t ) }-e^{i\lambda\beta ( x_{0}-t ) } \bigr] \,dt \biggr] \\ &{}+\frac{\alpha_{2}}{4}\biggl[ e^{2i\lambda\beta x_{0}}+1+P ( x_{0},x_{0} ) \frac{e^{2i\lambda\beta x_{0}}}{i\lambda\beta}-\frac{P ( x_{0},x_{0} ) }{i\lambda\beta} \\ & +\frac{1}{i\lambda\beta} \int_{0}^{x_{0}}P_{x} ( x_{0},t ) \bigl[ e^{i\lambda\beta ( x_{0}+t ) }-e^{i\lambda \beta ( x_{0}-t ) } \bigr] \,dt\biggr] \\ &{}+\frac{\alpha_{3}}{4\beta} \biggl[ \frac{e^{2i\lambda\beta x_{0}}}{\lambda^{2}}- \frac{1}{\lambda^{2}}+ \frac{1}{\lambda^{2}} \int_{0}^{x_{0}}P ( x_{0},t ) \bigl[ e^{i\lambda\beta ( x_{0}+t ) }-e^{i\lambda \beta ( x_{0}-t ) } \bigr] \,dt \biggr] \\ &{}+\frac{i\alpha_{4}}{4}\biggl[ \frac{e^{2i\lambda\beta x_{0}}}{\lambda }+\frac{1}{ \lambda}+P ( x_{0},x_{0} ) \frac{e^{2i\lambda\beta x_{0}}}{ i\beta\lambda^{2}}-\frac{P ( x_{0},x_{0} ) }{i\beta\lambda^{2} } \\ &{} +\frac{1}{i\beta\lambda^{2}} \int_{0}^{x_{0}}P_{x} ( x_{0},t ) \bigl[ e^{i\lambda\beta ( x_{0}+t ) }-e^{i\lambda\beta ( x_{0}-t ) } \bigr] \,dt\biggr] \biggr\} \\ =&e^{i\lambda x_{0} ( 1-\beta ) } \biggl\{ O \biggl( \frac{1}{ \lambda} \biggr) + \frac{\alpha_{2}}{4}+O \biggl( \frac{1}{\lambda} \biggr) +O \biggl( \frac{1}{\lambda^{2}} \biggr) +O \biggl( \frac{1}{\lambda} \biggr) \biggr\} \\ =&e^{i\lambda x_{0} ( 1-\beta ) }\frac{\alpha_{2}}{4} \biggl[ 1+O \biggl( \frac{1}{\lambda} \biggr) \biggr] . \end{aligned}$$

This completes the proof of (3.1). Similarly, (3.2) can be proved easily. □

In this section, we assume that $\beta\geq1 $. We give a lemma, which is necessary to discuss the properties of eigenvalues and spectral singularities of *L*.

### Lemma 3.2

*Assume* (2.4).

(i) *The set* $S_{1} $ *is bounded*, *and no more than a countable number of elements and its limit points can lie on a bounded subinterval of the real axis*.
(ii) *The set* $S_{2} $ *is compact*, *and its linear Lebesgue measure is zero*.

### Proof

Asymptotic equation (3.1) shows that $M_{22} $ cannot equal zero for sufficiently large $\lambda\in\overline{C}_{+} $. Thus the boundedness of the sets $S_{1} $ and $S_{2} $ follows from (3.1). Moreover, since $M_{22} $ is analytic in $\mathbb{C}_{+} $, the set $S_{1} $ has at most countable number of elements, and its limit points can lie only on a bounded subinterval of the real axis. Using the uniqueness theorem of analytic functions [23], we obtain that $S_{2} $ is a closed set and its linear Lebesgue measure is zero. □

Now, we can give the following theorem.

### Theorem 3.3

*Under condition* (2.4),

(i) *The set of eigenvalues of* *L* *is bounded and has at most a countable number of elements*, *and its limit points can lie only on a bounded subinterval of the real axis*.
(ii) *The set of spectral singularities of* *L* *is compact*, *and its linear Lebesgue measure is zero*.

Now, we proceed by assuming an extra condition on *q* to assure the finiteness of the sets $\sigma_{d}(L) $ and $\sigma_{ss}(L) $.

### Theorem 3.4

*If*

3.4 $$ \int_{0}^{\infty} \exp(\epsilon x) \bigl\vert q(x) \bigr\vert \,dx< \infty $$

*for every* $\epsilon>0 $, *then there are finitely many eigenvalues and spectral singularities of the operator* *L*, *and each of them has finite multiplicity*.

### Proof

Using (2.9), (2.10), and (3.4), we find that

$$\bigl\vert K(x_{0},t) \bigr\vert \leq c\exp \biggl( -t \frac{\epsilon }{2} \biggr) ,\qquad \bigl\vert K_{x}(x_{0},t) \bigr\vert \leq c\exp \biggl( -t\frac{\epsilon}{2} \biggr) , $$

that is, the function $M_{22} $ has an analytic continuation from the real axis to the lower half-plane $\operatorname{Im} \lambda>-\epsilon /2 $. Hence the sets $\sigma_{d}(L) $ and $\sigma_{ss}(L) $ have no limit points on the real line, and by Theorem 3.3 these sets are bounded and have a finite number of elements. Finally, using the uniqueness theorem of analytic functions [23], we see that all zeros of $M_{22} $ in $\overline {C}_{+} $ have finite multiplicities. □

Now, let us denote the set of all limit points of $S_{1} $ by $S_{3} $ and the set of all zeros of $M_{22} $ with infinite multiplicity in $\overline{C}_{+} $ by $S_{4} $. By the uniqueness theorem of analytic functions, we find that

3.5 $$ S_{1}\cap S_{4}=\emptyset, \qquad S_{3}\subset S_{2},\qquad S_{4}\subset S_{2},\qquad \mu(S_{3})=0,\qquad\mu(S_{4})=0. $$

From the continuity of all derivatives of $M_{22} $ up to the real axis, we obtain that

3.6 $$ S_{3}\subset S_{4}. $$

Next, we indicate the same result of Theorem 3.4 by using a weaker condition than (3.4).

### Theorem 3.5

*If*

3.7 $$ \int_{0}^{\infty}\exp \bigl( \epsilon x^{\delta}\bigr) \bigl\vert q(x) \bigr\vert \,dx < \infty $$

*for some* $\epsilon>0$ *and* $\frac{1}{2}\leq\delta<1 $, *then*

3.8 $$ S_{4}=\emptyset. $$

### Proof

Since $M_{22} $ cannot be continued analytically from the real line to the lower half-plane under condition (3.7), it is not possible to prove the finiteness of eigenvalues and spectral singularities in a way similar to Theorem 3.4.

On the other hand, from (2.15) we have

3.9 $$ \begin{aligned}[b] \lambda M_{22}(\lambda)&=\frac{i}{2} \bigl[ \alpha_{4} M_{1}(\lambda )M_{3}(\lambda)+ \alpha_{3} M_{1}(\lambda)M_{4}(\lambda)\\ &\quad {}- \alpha_{2} M_{2}(\lambda )M_{3}(\lambda)- \alpha_{1}M_{2}(\lambda)M_{4}(\lambda) \bigr], \end{aligned} $$

where

$$\begin{aligned}& M_{1}(\lambda) = 1+ \int_{x_{0}}^{\infty}K(x_{0},t)e^{i\lambda(t-x_{0})}\,dt, \\& M_{2}(\lambda) = i\lambda-K(x_{0},x_{0})+ \int_{x_{0}}^{\infty }K_{x}(x_{0},t)e^{i\lambda(t-x_{0})}\,dt, \\& M_{3}(\lambda) = (\cos\lambda\beta x_{0}) e^{i\lambda x_{0}}+P(x_{0},x_{0})e^{i\lambda x_{0}} \frac{\sin\lambda\beta x_{0}}{\lambda \beta}+ \int_{0}^{x_{0}}P_{x}(x_{0},t)e^{i\lambda x_{0}} \frac{\sin\lambda\beta t}{\lambda\beta}\,dt, \\& M_{4}(\lambda) = e^{i\lambda x_{0}}\frac{\sin\lambda\beta x_{0}}{\lambda \beta}+ \int_{0}^{x_{0}}P(x_{0},t)e^{i\lambda x_{0}} \frac{\sin\lambda\beta t}{\lambda\beta}\,dt. \end{aligned}$$

Moreover, it follows from (2.9), (2.10), and (2.23) that $M_{22} $ is analytic in $\mathbb{C}_{+} $ and all of its derivatives are continuous up to the real axis. Then, using (2.9), (2.10), and (3.7), we obtain

3.10 $$ \biggl\vert \frac{d^{n}}{d\lambda^{n}}M_{i}(\lambda) \biggr\vert \leq c_{1} \int _{x_{0}}^{\infty} \bigl[ (1+\beta)t \bigr] ^{n} \exp \biggl( -\epsilon \biggl( \frac{t}{2} \biggr) ^{\delta}\biggr) \,dt,\quad i=1,2, $$

where $\lambda\in\mathbb{C}_{+} $, $\vert \lambda \vert < H $, and $m=0,1,2,\ldots $ . From the continuity of the functions *P* and $P_{x} $ we get

3.11 $$ \biggl\vert \frac{d^{n}}{d\lambda^{n}}M_{i}(\lambda) \biggr\vert \leq c_{2} \bigl[ (1+\beta)x_{0} \bigr] ^{n}, \quad i=3,4, $$

where $\lambda\in\mathbb{C}_{+} $, $\vert \lambda \vert < H $, and $m=0,1,2,\ldots $. Thus, from (3.9)–(3.11) we have

3.12 $$\begin{aligned} \biggl\vert \frac{d^{n}}{d\lambda^{n}}(\lambda M_{22}) \biggr\vert & \leq {\sum_{s=0}^{n}} \left ( \begin{matrix} n\\ s \end{matrix} \right ) \biggl\vert \frac{d^{n-s}}{d\lambda^{n-s}}M_{1}(\lambda) \biggr\vert \biggl[ \vert \alpha_{4} \vert \biggl\vert \frac{d^{s}}{d\lambda ^{s}}M_{3}( \lambda) \biggr\vert + \vert \alpha_{3} \vert \biggl\vert \frac{d^{s} }{d\lambda^{s}}M_{4}(\lambda) \biggr\vert \biggr] \\ &\quad {} + {\sum_{s=0}^{n}} \left ( \begin{matrix} n\\ s \end{matrix} \right ) \biggl\vert \frac{d^{n-s}}{d\lambda^{n-s}}M_{2}(\lambda) \biggr\vert \biggl[ \vert \alpha_{2} \vert \biggl\vert \frac{d^{s}}{d\lambda^{s}} M_{3}( \lambda) \biggr\vert + \vert \alpha_{1} \vert \biggl\vert \frac{d^{s} }{d\lambda^{s}}M_{4}(\lambda) \biggr\vert \biggr] \\ & \leq c_{3}\alpha {\sum_{s=0}^{n}} \left ( \begin{matrix} n\\ s \end{matrix} \right ) { \int_{x_{0}}^{\infty}} \bigl[ (1+\beta)x_{0} \bigr] ^{s} \bigl[ (1+\beta)t \bigr] ^{n-s} \exp \biggl( - \epsilon \biggl( \frac{t}{2} \biggr) ^{\delta} \biggr) \,dt \\ & \leq c_{3}\alpha2^{n} (1+\beta)^{n} { \int_{0}^{\infty}} t^{n}\exp \biggl( -\epsilon \biggl( \frac{t}{2} \biggr) ^{\delta} \biggr) \,dt \end{aligned}$$

for $\alpha:=( \vert \alpha_{1} \vert + \vert \alpha_{2} \vert + \vert \alpha_{3} \vert + \vert \alpha_{4} \vert ) $ and $n=1,2,\dots$. Now we can write

$$\biggl\vert \frac{d^{n}}{d\lambda^{n}}(\lambda M_{22}) \biggr\vert \leq A_{n} $$

for $n=1,2,\ldots $ and $\lambda\in\mathbb{C}_{+} $, $\vert \lambda \vert < H $, where

$$A_{n}=c_{3}\alpha2^{n} (1+\beta)^{n} { \int_{0}^{\infty}} t^{n}\exp \biggl( -\epsilon \biggl( \frac{t}{2} \biggr) ^{\delta} \biggr) \,dt . $$

Since $M_{22} $ is cannot be zero, it follows from Pavlov’s theorem [24] that

3.13 $$ \int_{0}^{h}\ln T(s)\,d\mu(S_{4},s)>- \infty, $$

where $T(s)=\inf \lbrace\frac{A_{n}s^{n}}{n!}:n=0,1,2,\ldots \rbrace $, and $\mu(S_{4},s) $ is the linear Lebesgue measure of the *s*-neighborhood of $S_{4} $. Also, for $A_{n} $, using the gamma function, we can write

3.14 $$ A_{n} \leq K\alpha b^{n} n^{n(1-\delta)/\delta}n!, $$

where *K* and *b* are constants depending on $c_{3} $, *ϵ*, *β*, and *δ*. Therefore it is obvious that

3.15 $$ T(s)\leq K\alpha\exp \biggl\lbrace {-\frac{1-\delta}{\delta }}e^{-1}b^{-\delta/(1-\delta)}s^{-\delta/(1-\delta)} \biggr\rbrace . $$

From (3.13) and (3.15) we get

3.16 $$ \int_{0}^{h}s^{-\delta/(1-\delta)}\,d\mu(S_{4},s)< \infty. $$

Since $\delta/(1-\delta)\geq1$, it follows from (3.16) that $\mu (S_{4},s) =0 $, that is, $S_{4}=\emptyset$. □

### Theorem 3.6

*Assume* (3.7). *Then the operator* *L* *has a finite number of eigenvalues and spectral singularities*, *and each of them is of finite multiplicity*.

### Proof

Using Lemma 3.2, (3.6), and (3.8), we obtain that $S_{3}=\emptyset$ and the sets $S_{1} $ and $S_{2} $ are countable and bounded. Then it follows from (3.8) that the spectral singularities and eigenvalues of *L* have finite multiplicities. □

We remark that, by the help of asymptotic equation (3.2), similar results can be given for $\beta\leq-1 $.

## Scattering function of the impulsive operator

In this section, we determine the scattering function of the impulsive Sturm–Liouville operator *L*.

### Theorem 4.1

*Let* $\alpha_{1}, \alpha_{2}, \alpha_{3}, \alpha_{4}\in\mathbb{R} $, $\beta\in \mathbb{R}\backslash(-1,1) $, *and* $\lambda\in\mathbb{R} \backslash \{ 0 \} $. *Then* $M_{22} ( \lambda ) \neq0$.

### Proof

Let us consider the solutions *F* and *G* of (2.1)–(2.3) defined by expressions (2.17) and (2.18) for $\alpha_{1}, \alpha _{2}, \alpha_{3}, \alpha_{4}\in\mathbb{R} $ and $\beta\in\mathbb {R}\backslash(-1,1) $, respectively. Then it follows from (2.15), (2.16), and (2.20) that

4.1 $$ \overline{B_{+}^{-}}=\overline {M_{22} ( \lambda ) }=M_{12} ( \lambda )=A_{+}^{-} ,\quad\lambda\in\mathbb{R} \backslash \{ 0 \}. $$

Assume that, for any real nonzero $\lambda_{0} $ such that $M_{22} ( \lambda_{0} )=0$. This gives that

$A_{+} ^{-} ( \lambda_{0} ) =B_{+}^{-} ( \lambda_{0} ) =0$ by (4.1). In this case, $G ( x,\lambda _{0} ) $ turns into a trivial solution of (2.1)–(2.3), which gives a contradiction with our assumption, that is, $M_{22} ( \lambda ) \neq0$ for all $\lambda\in\mathbb{R} \backslash \{ 0 \} $, $\beta\in\mathbb{R}\backslash(-1,1) $, and $\alpha_{1}, \alpha_{2}, \alpha_{3}, \alpha_{4} \in\mathbb{R} $. □

### Corollary 4.2

*Let* $\alpha_{1}, \alpha_{2}, \alpha_{3}, \alpha_{4}\in\mathbb{R} $ *and* $\beta \in\mathbb{R}\backslash(-1,1) $. *The operator* *L* *has no spectral singularities*.

### Definition 4.3

Let $\alpha_{1}, \alpha_{2}, \alpha_{3}, \alpha_{4}\in\mathbb{R} $ and $\beta \in\mathbb{R}\backslash(-1,1) $. Then the scattering function of the operator *L* is defined by

$$\mathcal{S} ( \lambda ) =\frac{F ( 0,-\lambda ) }{F ( 0,\lambda ) }. $$

Since *q* is a real-valued potential function, it is evident from (2.17) and (2.20) that

$$\overline{F ( x,\lambda ) }=F ( x,-\lambda ) $$

for all $\lambda\in\mathbb{R} \backslash \{ 0 \} $. Then the definition of the function $\mathcal{S} $ turns into

4.2 $$ \mathcal{S} ( \lambda ) =\frac{\overline{F ( 0,\lambda ) }}{F ( 0,\lambda ) }=\frac{\overline{M_{22} ( \lambda ) }}{ M_{22} ( \lambda ) } = \frac{M_{12} ( \lambda ) }{M_{22} ( \lambda ) },\quad\lambda\in\mathbb{R} \backslash \{ 0 \}. $$

From (2.15), (2.16), and (4.2), it is clear that, for all $\lambda\in\mathbb{R} \backslash \{ 0 \} $,

$$\mathcal{S} ( \lambda ) =\frac{e' ( x_{0},-\lambda ) ( \alpha_{1} S ( x_{0},\lambda^{2} ) +\alpha_{2} S' ( x_{0},\lambda^{2} ) ) -e ( x_{0},-\lambda ) ( \alpha_{3} S ( x_{0},\lambda^{2} ) +\alpha_{4} S' ( x_{0},\lambda ^{2} ) ) }{-e' ( x_{0},\lambda ) ( \alpha_{1} S ( x_{0},\lambda^{2} ) +\alpha_{2} S' ( x_{0},\lambda ^{2} ) ) +e ( x_{0},\lambda ) ( \alpha_{3} S ( x_{0},\lambda^{2} ) +\alpha_{4} S' ( x_{0},\lambda^{2} ) ) }. $$

### Theorem 4.4

*Let* $\alpha_{1}, \alpha_{2}, \alpha_{3}, \alpha_{4} \in\mathbb{R} $ *and* $\beta \in\mathbb{R}\backslash(-1,1) $. *For all* $\lambda\in\mathbb{R} \backslash \{ 0 \} $, *the scattering function yields*

$$\mathcal{S} ( -\lambda ) =\mathcal{S}^{-1} ( \lambda ) =\overline{ \mathcal{S} (\lambda ) }. $$

### Proof

By (4.2) we obtain

4.3 $$ \mathcal{S} ( -\lambda ) =\frac{{M_{12} ( -\lambda ) }}{M_{22} ( -\lambda ) }. $$

Since $\overline{M_{22} ( -\lambda ) }=M_{22} ( \lambda ) $ and $\overline{M_{12} ( -\lambda ) }=M_{12} ( \lambda ) $ for all $\lambda\in\mathbb{R} \backslash \{ 0 \} $, $\beta\in\mathbb{R}\backslash(-1,1) $, and $\alpha_{1}, \alpha_{2}, \alpha_{3}, \alpha_{4} \in\mathbb{R} $, we get

$$\mathcal{S} ( -\lambda ) =\mathcal{S}^{-1} ( \lambda ) =\overline{ \mathcal{S} (\lambda ) }. $$

The proof is completed. □

## An example

Let us consider the Sturm–Liouville operator $L_{0} $ in $L^{2}[0,\infty ) $ created by the following impulsive problem:

5.1 $$ \textstyle\begin{cases} -y''=\lambda^{2}\rho ( x ) y,\quad x\in{[0,x_{0})}\cup ( x_{0},\infty ), \\ y ( 0 ) =0, \\ \bigl[ {\scriptsize\begin{matrix}{} y ( x_{0}^{+} ) \cr y' ( x_{0}^{+} ) \end{matrix}}\bigr] =B \bigl[ {\scriptsize\begin{matrix}{} y ( x_{0}^{-} ) \cr y' ( x_{0}^{-} ) \end{matrix}}\bigr] ,\quad B= \bigl[ {\scriptsize\begin{matrix}{} \alpha_{1} & 0 \cr 0 & \alpha_{4} \end{matrix}}\bigr] , \end{cases} $$

where *ρ* is the density function given by

$$\rho ( x ) = \textstyle\begin{cases} \beta^{2},& 0\leq x< x_{0},\\ 1,& x>x_{0}, \end{cases} $$

such that $\beta\in\mathbb{C}\backslash \{ 0 \} $, $\alpha _{1},\alpha_{4}\in\mathbb{C} $, $\alpha_{1}.\alpha_{4}\neq0$, and $x_{0}\in \mathbb{R} ^{+}$. Using $q=0 $ in (2.23), we directly obtain

5.2 $$ M_{22} ( \lambda ) =\frac{i}{2\lambda}e^{i\lambda x_{0}} \biggl[ -\frac{i\alpha_{1}}{\beta}\sin\lambda\beta x_{0}+\alpha_{4} \cos\lambda\beta x_{0} \biggr]. $$

To investigate the eigenvalues and spectral singularities of $L_{0} $, we examine the zeros of $M_{22} $. For this purpose, we see that

$$e^{2i\lambda\beta x_{0}}=\frac{\alpha_{1}+\beta\alpha_{4}}{\alpha_{1}-\beta \alpha_{4}} $$

by (5.2). Using the last equation, we find

5.3 $$ \lambda_{k}=-\frac{i}{2\beta x_{0}}\ln \biggl\vert \frac{1+A}{1-A} \biggr\vert +\frac{1}{2\beta x_{0}} \biggl[ \operatorname{Arg} \biggl( \frac {1+A}{1-A} \biggr) +2k\pi \biggr] ,\quad k\in \mathbb{Z}, $$

where $A=\frac{\beta\alpha_{4}}{\alpha_{1}}$.

Let $\beta=a+ib$. Then we can write real and imaginary parts of $\lambda_{k}$ by

5.4 $$ \operatorname{Re} \lambda_{k} =\frac{1}{2x_{0} \vert \beta \vert ^{2} } \biggl\lbrace a \biggl[ \operatorname{Arg} \biggl( \frac{1+A}{1-A} \biggr) +2k\pi \biggr] -b \ln \biggl\vert \frac{1+A}{1-A} \biggr\vert \biggr\rbrace $$

and

5.5 $$ \operatorname{Im} \lambda_{k} =-\frac{1}{2x_{0} \vert \beta \vert ^{2}} \biggl\lbrace a\ln \biggl\vert \frac{1+A}{1-A} \biggr\vert +b \biggl[ \operatorname{Arg} \biggl( \frac{1+A}{1-A} \biggr) +2k\pi \biggr] \biggr\rbrace , $$

respectively. It is easy to see that if

$$\biggl[ a\ln \biggl\vert \frac{1+A}{1-A} \biggr\vert +b \biggl( \operatorname {Arg} \biggl( \frac{1+A}{1-A} \biggr) +2k\pi \biggr) \biggr] =0, $$

then the problem has spectral singularities, and if

5.6 $$ \biggl[ a\ln \biggl\vert \frac{1+A}{1-A} \biggr\vert +b \biggl( \operatorname{Arg} \biggl( \frac{1+A}{1-A} \biggr) +2k\pi \biggr) \biggr] < 0, $$

then the problem has eigenvalues. Now, we investigate some particular cases.

*Case1*: Let $A=\frac{e^{i\theta}-1}{e^{i\theta}+1}$ for $\theta\in \mathbb{R}$. In this case, since $\operatorname{Arg} ( \frac{1+A}{1-A} )=\theta$, we get

$$\lambda_{k}=\frac{\theta+2k\pi}{2\beta x_{0}},\quad k \in\mathbb{Z}. $$

*1a*: Let $\beta\in\mathbb{R} $. Thus $\lambda_{k}\in\mathbb{R} $, and then the numbers $\mu_{k}=\lambda _{k}^{2}$, $k\in \mathbb{Z} $, are the spectral singularities of the impulsive boundary value problem (5.1).

*1b*: Let $\beta\in \mathbb{C}$. We get

$$\operatorname{Im}\lambda_{k}=-\frac{1}{2x_{0} \vert \beta \vert ^{2} } \bigl[ b ( \theta+2k \pi ) \bigr],\quad k \in\mathbb{Z}, $$

by (5.5). If $b ( \theta+2k\pi )<0$, then the operator $L_{0} $ has eigenvalues. Otherwise, the problem has no eigenvalues and spectral singularities.

*Case2*: Let $\operatorname{Im} A \neq0 $. We investigate some subcases.

*2a*: Let *A* be purely imaginary, that is, $\operatorname{Re} A=0 $. In this case, since $\vert \frac{1+A}{1-A} \vert =1$, from (5.3) we get

$$\lambda_{k}=\frac{\operatorname{arg}(1+A)}{\beta x_{0}},\quad k\in\mathbb{Z}. $$

which means that the numbers $\mu_{k}=\lambda_{k}^{2}$, $k\in \mathbb{Z}$, are the spectral singularities of (5.1) for $\beta \in\mathbb{R} $.

Let $\beta\in\mathbb{C}$. We find

$$\operatorname{Im}\lambda_{k}=-\frac{b}{2x_{0} \vert \beta \vert ^{2} } \bigl[ \operatorname{arg}(1+A) \bigr], \quad k\in\mathbb{Z}. $$

If $b [ \operatorname{arg}(1+A) ] <0$, then the operator $L_{0} $ has eigenvalues. Otherwise, the operator has no eigenvalues and spectral singularities.

*2b*: Let $\operatorname{Re} A<0$. For $\beta\in\mathbb{R}$, that is, $b=0 $, we have

$$\operatorname{Im}\lambda_{k}=-\frac{1}{2x_{0}a } \biggl( \ln \biggl\vert \frac{1+A}{1-A} \biggr\vert \biggr), \quad k\in \mathbb{Z}. $$

If $a>0$, then the numbers $\mu_{k}=\lambda_{k}^{2}$, $k\in\mathbb {Z}$, are the eigenvalues of impulsive problem (5.1); otherwise, the operator $L_{0} $ has no eigenvalues.

Let $\beta\in\mathbb{C}$. If $a>0$ and $b ( \operatorname{Arg} ( \frac{1+A}{1-A} ) +2k\pi ) <0$, then there exist eigenvalues, and if $a<0$ and $b ( \operatorname{Arg} (\frac{1+A}{1-A} ) +2k\pi ) >0$, then the problem has no eigenvalues by (5.6).

*2c*: Let $\operatorname{Re} A>0$. In this case, since $\vert \frac{1+A}{1-A} \vert >1$, for $\beta\in\mathbb{R}$, we obtain

$$\operatorname{Im}\lambda_{k}=-\frac{1}{2x_{0}a } \biggl( \ln \biggl\vert \frac{1+A}{1-A} \biggr\vert \biggr), \quad k\in \mathbb{Z}. $$

If $a<0$, then the numbers $\mu_{k}=\lambda_{k}^{2}$, $k\in\mathbb {Z}$, are the eigenvalues of $L_{0} $; otherwise, the problem has no eigenvalues.

For $\beta\in\mathbb{C}$, if $a<0$ and $b ( \operatorname {Arg} ( \frac{1+A}{1-A} ) +2k\pi ) <0$, then there exist eigenvalues, and if $a>0$ and $b ( \operatorname{Arg} ( \frac{1+A}{ 1-A} ) +2k\pi ) >0$, then the problem has no eigenvalues.

*Case3*: Let *A* be a real number.

*3a*: Let $0< A<1$. In this case, we get

$$\lambda_{k}=-\frac{i}{2\beta x_{0}}\ln \biggl( \frac{1+A}{1-A} \biggr) +\frac{k\pi}{\beta x_{0}} ,\quad k\in \mathbb{Z}. $$

Let $\beta\in\mathbb{R}$. If $a<0$, then the numbers $\mu_{k}=\lambda _{k}^{2}$, $k\in \mathbb{Z}$, are the eigenvalues of (5.1). Otherwise, the problem has no eigenvalues. For $\beta\in\mathbb{C}$, we obtain

$$\operatorname{Im} \lambda_{k}=-\frac{1}{2x_{0} \vert \beta \vert ^{2} } \biggl[ a\ln \biggl( \frac{1+A}{1-A} \biggr) +b ( 2k\pi ) \biggr], \quad k\in\mathbb{Z}. $$

If $a<0$ and $b ( 2k\pi ) <0$, then we have eigenvalues of $L_{0} $, and if $a>0$ and $b ( 2k\pi ) >0$, then the problem has no eigenvalues.

*3b*: Let $1< A<\infty$. We find

$$\lambda_{k}=-\frac{i}{2\beta x_{0}}\ln \biggl\vert \frac{1+A}{1-A} \biggr\vert +\frac{1}{2\beta x_{0}} \bigl[\pi ( 2k+1 )\bigr], \quad k\in\mathbb{Z}. $$

For $\beta\in\mathbb{R}$, if $a<0$, then the numbers $\mu_{k}=\lambda _{k}^{2}$, $k\in \mathbb{Z}$, are the eigenvalues of impulsive problem (5.1). Otherwise, the problem has no eigenvalues. For $\beta\in \mathbb{C}$, if $a<0$ and

$b(2k+1)\pi<0$, then there exist eigenvalues, and if $a>0$ and $b (2k+1) \pi >0$, then there are no eigenvalues.

*3c*: Let $-1< A<0$. In this case, for $\beta\in\mathbb{R}$, if $a>0$, then the numbers $\mu_{k}=\lambda_{k}^{2}$, $k\in\mathbb {Z}$, are the eigenvalues of $L_{0} $. Otherwise, the problem has no eigenvalues. Let $\beta\in\mathbb{C}$. If $a>0$ and $b ( 2k \pi ) <0$, then the problem has eigenvalues. If $a<0$ and $b ( 2k\pi ) >0$, then the problem has no eigenvalues.

*3d*: Let $-\infty< A<-1$. In this case, for $\beta\in\mathbb {R}$, if $a>0$, then the numbers $\mu_{k}=\lambda_{k}^{2}$, $k\in\mathbb {Z}$, are the eigenvalues of (5.1); otherwise, the problem has no eigenvalues. For $\beta\in\mathbb{C}$, if $a>0$ and $b ( 2k+1 ) \pi<0$, then there exist eigenvalues. If $a<0$ and $b ( 2k+1 ) \pi>0$, then the problem has no eigenvalues.

*Case 4*: Let *β* be purely imaginary, that is, $a=0$. In this case, we find

$$\operatorname{Im}\lambda_{k}=-\frac{1}{2bx_{0} } \biggl[ \operatorname{Arg} \biggl( \frac{1+A}{1-A} \biggr) +2k\pi \biggr] ,\quad k\in \mathbb{Z}. $$

If $\operatorname{Arg} ( \frac{1+A}{1-A} ) +2k\pi=0$, then there exist spectral singularities of $L_{0} $.

If $b [ \operatorname{Arg} ( \frac{1+A}{1-A} ) +2k\pi ] <0 $, then the numbers $\mu_{k}=\lambda_{k}^{2}$, $k\in\mathbb{Z}$, are the eigenvalues of $L_{0} $; otherwise, the problem has no eigenvalues.

## Conclusions

In this study, we discuss some spectral and scattering problems of an impulsive Sturm–Liouville boundary value problem on the semi axis. Although there are various studies about the spectral analysis of these problems, much of them are on the whole axis. Moreover, the method we use to investigate the eigenvalues and spectral singularities is quite different from other papers. By using a transfer matrix we introduce the sets of eigenvalues and spectral singularities, and under sufficient conditions, we guarantee the finiteness of these sets.
